# Intercultural sensitivity, challenges, and perceived value in multicultural group work among third-year medical students at Alexandria university, Egypt (2023–2024)

**DOI:** 10.1186/s12909-025-07597-7

**Published:** 2025-07-11

**Authors:** Marium Mahmoud Abdulla, Aida Mohey Mohamed Ali

**Affiliations:** Department of Community Medicine and Public Health, Faculty of Medicine, Alexandria University, Azarita, Champollion Street, Alexandria, 21521 Egypt

**Keywords:** Intercultural sensitivity, Multicultural student group work challenges, Diversity, Perceived value, Domestic, International, Medical students

## Abstract

**Background:**

With the advent of globalization, intercultural sensitivity (ICS) is a vital skill, particularly for medical students. At the Alexandria Faculty of Medicine (AFM), third-year domestic (Egyptian) and international students in the national program have been separated throughout all the years due to logistical challenges. This study aimed to assess the ICS of third-year national program students, identify its correlates, explore multicultural student groups (MCSG) work-related challenges, and evaluate the perceived value of multicultural group work.

**Methods:**

A cross-sectional study using quota-convenience sampling was conducted among third-year national program medical students at AFM to proportionally represent domestic and international students. Participants completed an online questionnaire covering: general characteristics, the Arabic-translated version of: the Intercultural Sensitivity Scale (ISS), MCSG work-related challenges, and perceived value of intercultural group work. Statistical analyses included Mann-Whitney, Kruskal-Wallis, and Spearman’s tests to examine associations between ICS and various factors.

**Results:**

Among the 272 participants (170 domestic, 102 international), generally high levels of ICS were reported. Higher ICS was significantly associated with being an international student, higher English proficiency, multilingualism, and prior intercultural exposure (*P* < 0.05). Domestic students reported more MCSG-related challenges and a greater perceived value in multicultural group work (*P* < 0.05).

**Conclusion:**

Third-year AFM students reported high ICS overall. Domestic students anticipated more MCSG work-related challenges yet perceived greater value in intercultural group work. Given the non-random sampling, findings may not be generalizable beyond the study population. The educational institution should foster opportunities for intercultural interactions among students, both within and outside the classroom.

**Supplementary Information:**

The online version contains supplementary material available at 10.1186/s12909-025-07597-7.

## Background

Globalization is one of the most significant transformations occurring in the world today, making exposure to diverse cultures unavoidable [1]. As a result, it is essential for university graduates to develop certain skills to be prepared for the 21st century workforce. One such crucial skill is intercultural sensitivity (ICS), defined as “an individual’s ability to develop a positive emotion towards understanding and appreciating cultural differences that promotes appropriate and effective behavior in intercultural communication” [2]. Given its importance, ICS is especially critical in healthcare settings, benefiting both patients [3] and healthcare providers [4, 5].

Medical students, much like other university students, engage in group work and collaborative assignments. When these assignments involve participants from various cultural backgrounds, they present both challenges and opportunities for intercultural learning, provided that students recognize the value of multicultural education [6, 7].

At the Alexandria Faculty of Medicine (AFM), the National program comprises both domestic (Egyptian) and international (non-Egyptian) students. Traditionally, students from these groups would start their studies separately and integrate in their third year. However, the class of 2021–2026 did not experience this integration due to logistical issues, leaving domestic students without any interactions in multicultural student groups (MCSG).

To date, there has been only one study published on the ICS of Arab university students [8], and none specifically addressing Arab medical students’ ICS. Therefore, this study aimed to evaluate the ICS of third-year national program medical students at AFM (objective 1), identify its correlates (objective 2), explore the MCSG work-related challenges (objective 3), assess the perceived value of intercultural group work (objective 4) and analyze the relationships between.

ICS and MCSG work-related challenges (objective 5) and MCSG work-related challenges and the perceived value of intercultural group work (objective 6).

## Methods

This cross-sectional study used quota-convenience sampling method to ensure proportional representation of domestic and international students among third-year national program medical students at AFM, with two-thirds being domestic. While this approach facilitated access to participants from both groups, it may have introduced selection bias and limits the generalizability of the findings to the wider student population. A total of 272 students participated, including 170 domestic and 102 international students. Older students retaking third-year classes were excluded. Data collection occurred over nearly three weeks, from December 4th to December 22nd 2023, during their academic year 2023–2024. Students were informed about the study through various channels, including class announcements, assignment meetings, office hours, and online groups. Those who agreed to participate completed an Arabic online questionnaire via Google Forms. Representatives from different student groups and nationalities were also contacted to disseminate the survey link. The online questionnaire consisted of four sections, with slight variations between domestic and international versions. English translations of both questionnaires are provided as supplementary files (Supplementary File 1: domestic version; Supplementary File 2: international version). The four sections were:

1. **General Characteristics**: Questions covered demographics, educational backgrounds, parental characteristics, overseas experiences and multicultural experiences.
2. **Intercultural Sensitivity Scale (ISS)**: The original scale consists of 24 items and has a reliability coefficient of 0.86 [9]. A culturally translated form by the researchers was used. The scale measures five subdimensions: interaction engagement, respect for cultural differences, interaction confidence, interaction enjoyment, and interaction attentiveness. Responses were recorded on a 5-point Likert scale (1 to 5), with reverse coding for specific items. Scores ranged from 24 to 120, with higher scores indicating greater ICS.
3. **Multicultural Student Group (MCSG) Work-Related Challenges**: A list of 17 potential challenges, based on literature [10], was translated into Arabic. Domestic students answered prospectively about expected challenges, while international students answered retrospectively about experienced challenges. Responses were on a 5-point Likert scale (1 to 5), with higher scores indicating greater challenges.
4. **Perceived Value of Intercultural Group Work**: Participants rated their agreement with four statements (translated to Arabic) about the value of intercultural group work on a 5-point Likert scale (1 to 5), with higher scores indicating greater perceived value of intercultural group work. Domestic students answered prospectively, while international students answered retrospectively. The original reliability of this section was good, with a Cronbach’s Alpha of 0.79 [7].

The data collection tool was initially pre-tested on 11 third-year medical students from five different nationalities (Egyptian, Sudanese, Iraqi, Syrian, and Jordanian) to ensure clarity of the translation and to estimate the time required to complete the questionnaire. Based on their feedback, wording modifications were made. Those students were excluded from final analysis.

The internal consistency of the Arabic-translated version of ISS and perceived value in intercultural group work scales was assessed using Cronbach’s alpha. While the total ISS scale showed good reliability (α = 0.836), some subscales demonstrated suboptimal internal consistency, with Cronbach’s alpha values ranging from 0.443 to 0.794. The low reliability observed in some subdimensions, particularly interaction attentiveness and respect for cultural differences, indicates possible measurement limitations. As the pilot testing aimed primarily to ensure linguistic clarity and comprehension rather than full psychometric validation, statistical refinement of the subscales was beyond the scope of this study. However, the observed reliability concerns highlight the importance of future work focused on cultural and psychometric validation of the Arabic-translated ISS. This limitation is further discussed in the discussion section, along with recommendations for future validation efforts. In contrast, the perceived value in intercultural group work scale demonstrated high internal consistency (α = 0.865). Detailed reliability coefficients for all subscales are provided in Supplementary File 3.

Ethics approval was obtained from the Institutional Review Board (IRB No: 00012098) at the Faculty of Medicine, Alexandria University. Permissions to collect data from students were granted by the Vice Dean for Student Affairs and Education. Additionally, permission to use and translate ISS into Arabic was secured via email from Prof. Guo-Ming Chen. A consent statement was included at the beginning of the form, declaring that by clicking “I agree” the participant acknowledged their agreement to participate in the study, on a voluntary basis, and that they could withdraw at any time. Informed consent was collected from all participants. Only those participants who voluntarily consented continued with the study. No identifiable data were collected, ensuring participant anonymity.

Sample size calculation was based on data from similar research showing that the mean total ISS score was 90.49 ± 12.68, for a sample size of 277 [11]. The formula employed here for sample size calculation was: Necessary sample size = Z^2^ SD^2^ / d^2^ [12]where: Z = 1.96, SD = Standard deviation, d = absolute precision or margin of error. Thus, the necessary sample size ≈ 270 students, proportionally stratified into 170 domestic and 100 international students.

Data analysis was conducted using the Statistical Package for Social Sciences (SPSS) version 24.0. Descriptive statistics are presented as mean ± SD, [95% CI for Mean] to align with common reporting practices in similar ICS studies, while inferential analyses used non-parametric tests (Mann-Whitney U, Kruskal-Wallis H, and Spearman’s tests) due to the data’s non-normal distribution. A significance level of (p *≤* 0.05) was applied to all analyses.

## Results

### General characteristics of the studied population

Supplementary file 4 presents participants’ background information. Most students were domestic (62.5%) and male (53.7%), with the majority educated in national schools (77.2%). About 47% reported good English proficiency, and 27% were plurilingual. Roughly one-third had lived abroad (35.7%) or traveled for tourism (37.5%). As for multicultural exposure: 80% had friends from different cultures, 49% had multicultural neighbors, and 62% had attended cultural events.

### Intercultural sensitivity scores and its correlates (objectives 1 and 2)

Table 1 presents the distribution of mean scores and 95% confidence intervals for each subdimension and the total ISS, stratified by key student characteristics. Overall, students demonstrated relatively high ICS scores, with a total mean of 90.13 (± 10.73), and higher scores in interaction engagement and respect for cultural differences.

**Table 1:** Distribution of intercultural sensitivity scale subdimensions and total scores based on students’ characteristics (*n* = 272)

Characteristic	**Interaction engagement**	Respect for	**Interaction confidence**	Interaction enjoyment	Interaction attentiveness	Total ICS score
		cultural differences				
Mean ± SD						
95% CI for Mean]						
**All participants**	28.07 ± 4.15	19.51 ± 2.98	18.57 ± 3.93	13.05 ± 2.10	10.93 ± 2.29	90.13 ± 10.73
(n = 272)	[7.57–28.56]	[19.16–19.87]	[18.10-19.04]	[12.80–13.30]	[10.66–11.20]	[88.85–91.41]
**Class** ^**$**^						
Domestic (n = 170)	27.58 ± 4.24	19.59 ± 2.825	17.69 ± 3.98	12.92 ± 1.99	10.66 ± 2.27	88.45 ± 10.88
	[26.94–28.23]	[19.16–20.02]	[17.09–18.30]	[12.62–13.23]	[10.32–11.01]	[86.80–90.10]
International (n = 102)	28.87 ± 3.88	19.39 ± 3.23	20.03 ± 3.73	13.26 ± 2.25	11.37 ± 2.27	92.93 ± 9.91
	[28.11–29.63]	[18.76–20.03]	[19.37–20.69]	[12.82–13.71]	[10.93–11.82]	[90.99–94.88]
	P= 0.019*	P= 0.566	P< 0.001*	P= 0.039*	P= 0.013*	P= 0.001*
**Gender** ^**$**^						
Male (n = 146)	27.83 ± 4.18	19.02 ± 3.01	18.99 ± 3.52	12.95 ± 2.14	11.18 ± 2.32	89.97 ± 10.53
	[27.14–28.51]	[18.53–19.51]	[18.42–19.57]	[12.60–13.30]	[10.80-11.56]	[88.25–91.70]
Female (n = 126)	28.34 ± 4.11	20.09 ± 2.85	18.08 ± 4.32	13.17 ± 2.05	10.64 ± 2.24	90.32 ± 10.96
	[27.62–29.07]	[19.58–20.59]	[17.32–18.84]	[12.81–13.53]	[10.25–11.04]	[88.38–92.25]
	P= 0.308	P= 0.001*	P= 0.110	P= 0.338	P= 0.60	P= 0.727
**Age** ^¶^	r= 0.056	r= -0.03	r= 0.188	r= 0.045	r= 0.184	r= 0.142
	P= 0.362	P= 0.618	P= 0.002*	P= 0.475	P= 0.002*	P= 0.042*
**Domestics’ residence** ^**$**^						
Urban (n = 129)	27.50 ± 4.12	19.54 ± 2.95	17.81 ± 3.83	12.94 ± 2.02	10.47 ± 2.22	88.27 ± 10.89
	[26.79–28.22]	[19.03–20.06]	[17.15–18.48]	[12.59–13.29]	[10.09–10.86]	[86.37–90.17]
Rural (n = 41)	27.83 ± 4.67	19.73 ± 2.43	17.32 ± 4.48	12.88 ± 1.95	11.27 ± 2.34	89.02 ± 10.96
	[26.36–29.30]	[18.96–20.50]	[15.90-18.73]	[12.26–13.49]	[10.53–12.01]	[85.57–92.48]
	P= 0.704	P= 0.968	P= 0.604	P= 0.783	P= 0.037*	P= 0.858
**Internationals’**						
**Nationality** ^**‡**^						
Jordanian (n = 28)	28.32 ± 4.43	18.36 ± 3.39	19.57 ± 3.18	12.50 ± 3.34	11.14 ± 2.38	89.89 ± 11.28
	[26.60-30.04]	[17.04–19.67]	[18.34–20.80]	[11.21–13.79]	[10.22–12.07]	[85.52–94.27]
Iraqi (n = 20)	27.95 ± 4.37	19.30 ± 3.67	20.65 ± 3.13	13.70 ± 1.46	11.80 ± 2.38	93.40 ± 11.66
	[25.90–30.00]	[17.58–21.02]	[19.18–22.12]	[13.02–14.38]	[10.69–12.91]	[87.95–98.85]
Palestinian (n = 16)	29.63 ± 3.58	19.44 ± 3.31	19.31 ± 3.83	13.56 ± 1.83	11.81 ± 1.97	93.75 ± 9.94
	[27.72–31.53]	[17.68–21.20]	[17.27–21.35]	[12.59–14.53]	[10.76–12.86]	[88.46–99.04]
Syrian (n = 15)	29.27 ± 3.79	20.07 ± 2.92	19.80 ± 3.49	13.27 ± 1.75	10.93 ± 2.58	93.33 ± 8.01
	[27.17–31.36]	[18.45–21.68]	[17.87–21.73]	[12.30-14.24]	[9.51–12.36]	[88.90-97.77]
Sudanese (n = 14)	29.64 ± 2.93	19.64 ± 2.65	19.93 ± 3.65	13.43 ± 1.39	11.36 ± 2.02	94.00 ± 7.54
	[27.95–31.33]	[18.11–21.17]	[17.82–22.03]	[12.62–14.24]	[10.19–12.53]	[89.65–98.35]
Yemeni (n = 4)	28.75 ± 4.03	19.75 ± 2.22	22.25 ± 2.22	14.50 ± 1.00	10.75 ± 2.63	96.00 ± 5.89
	[22.34–35.16]	[16.22–23.28]	[18.72–25.78]	[12.91–16.09]	[6.57–14.93]	[86.63-105.37]
Mauritanian (n = 4)	29.50 ± 2.38	22.25 ± 1.89	22.0 ± 4.24	13.00 ± 2.31	11.50 ± 2.89	98.25 ± 7.50
	[25.71–33.29]	[19.24–25.26]	[15.25–28.75]	[9.33–16.67]	[6.91–16.09]	[86.32-110.18]
Saudi^¥^ (n = 1)	32	23	20	15	11	101
	P= 0.865	P= 0.296	P= 0.592	P= 0.791	P= 0.900	P= 0.663
**International in touch with home culture** ^**$**^						
Yes (n = 100)						
	28.87 ± 3.91	19.36 ± 3.24	20.07 ± 3.38	13.24 ± 2.26	11.35 ± 2.29	92.89 ± 10.00
No (n = 2)	[28.09–29.65]	[18.72-20.00]	[19.40-20.74]	[12.79–13.69]	[10.89–11.81]	[90.91–94.87]
	29.00 ± 2.83	21.00 ± 2.83	18.00 ± 2.83	14.50 ± 0.71	12.50 ± 0.71	95.00 ± 1.41
	[3.59–54.41]	[-4.41-46.41]	[-7.41-43.41]	[8.15–20.85]	[6.15–18.85]	[82.29-107.71]
	P= 0.913	P= 0.489	P= 0.349	P= 0.449	P= 0.532	P= 0.839
**School system** ^**‡**^						
National (n = 210)	27.74 ± 4.24	19.45 ± 3.01	18.23 ± 3.97	12.91 ± 2.18	11.02 ± 2.20	89.35 ± 10.67
	[27.17–28.32]	[19.04–19.86]	[17.69–18.77]	[12.61–13.21]	[10.71–11.33]	[87.90–90.80]
International (n = 30)	29.30 ± 3.31	20.13 ± 2.78	19.70 ± 3.63	13.83 ± 1.29	10.37 ± 2.19	93.33 ± 10.18
	[28.06–30.54]	[19.09–21.17]	[18.34–21.06]	[13.35–14.31]	[9.55–11.18]	[89.53–97.14]
Mixed (n = 32)	29.03 ± 4.04	19.37 ± 2.98	19.75 ± 3.54	13.25 ± 2.03	10.88 ± 2.43	92.28 ± 11.13
	[27.75–30.49]	[18.30-20.45]	[18.47–21.03]	[12.52–13.98]	[10.00-11.75]	[88.27–96.29]
	P= 0.05*	P= 0.379	P= 0.035*	P= 0.091	P= 0.315	P= 0.054
**English proficiency** ^**‡**^						
Poor (n = 8)	28.75 ± 6.11	18.75 ± 2.49	16.75 ± 4.68	13.38 ± 0.92	11.13 ± 3.31	88.75 ± 14.34
	[23.64–33.86]	[16.67–20.83]	[12.84–20.66]	[12.61–14.14]	[8.35–13.90]	[76.76-100.74]
Moderate (n = 72)	27.75 ± 4.12	19.35 ± 3.13	17.82 ± 4.40	12.75 ± 2.41	11.10 ± 2.42	88.76 ± 10.51
	[26.78–28.72]	[18.61–20.08]	[16.79–18.85]	[12.18–13.32]	[10.53–11.66]	[86.30-91.23]
Good (n = 127)	27.69 ± 4.04	19.34 ± 2.94	18.46 ± 3.72	12.93 ± 2.05	10.87 ± 2.20	89.29 ± 10.61
	[26.98–28.40]	[18.82–19.85]	[17.80-19.11]	[12.57–13.29]	[10.49–11.26]	[87.43–91.15]
Excellent (n = 65)	29.06 ± 4.07	20.14 ± 2.91	19.85 ± 3.37	13.58 ± 1.84	10.83 ± 2.23	93.46 ± 10.31
	[28.05–30.07]	[19.42–20.86]	[19.01–20.68]	[13.13–14.04]	[10.28–11.38]	[90.91–96.02]
	P= 0.073	P= 0.216	P= 0.014*	P= 0.072	P= 0.840	P= 0.019*
**Plurilingualism** ^**$**^						
Yes (n = 73)	28.90 ± 4.10	19.52 ± 3.14	19.75 ± 3.96	13.22 ± 2.22	11.53 ± 2.35	92.93 ± 11.76
	[27.95–29.86]	[18.79–20.25]	[18.83–20.68]	[12.70-13.74]	[10.99–12.08]	[90.19–95.67]
No (n = 199)	27.76 ± 4.14	19.51 ± 2.93	18.14 ± 3.84	12.99 ± 2.05	10.71 ± 2.24	89.11 ± 10.17
	[27.18–28.34]	[19.10-19.92]	[17.60-18.67]	[12.70-13.28]	[10.40-11.02]	[87.68–90.53]
	P= 0.036*	P= 0.631	P= 0.002*	P= 0.170	P= 0.005*	P= 0.002*
**Parents’ background** ^**$**^						
Same (n = 259)	28.09 ± 4.12	19.51 ± 2.95	18.53 ± 3.92	13.04 ± 2.08	10.92 ± 2.27	90.09 ± 10.65
	[27.58–28.59]	[19.15–19.87]	[18.05–19.01]	[12.79–13.30]	[10.64–11.20]	[88.79–91.40]
Different (n = 13)	27.62 ± 4.68	19.54 ± 3.57	19.38 ± 4.13	13.23 ± 2.52	11.15 ± 2.74	90.92 ± 12.79
	[24.79–30.44]	[17.38–21.70]	[16.89–21.88]	[11.71–14.75]	[9.48–12.82]	[83.19–98.66]
	P= 0.807	P= 0.974	P= 0.444	P= 0.466	P= 0.576	P= 0.564
**Parents’ ICS** ^**‡**^						
Poor (n = 19)	28.53 ± 4.41	18.63 ± 2.41	18.68 ± 4.96	12.79 ± 2.39	11.47 ± 2.27	90.11 ± 11.93
	[26.40-30.65]	[17.47–19.79]	[16.30-21.07]	[11.64–13.94]	[10.38–12.57]	[84.36–95.85]
Average (n = 66)	27.08 ± 4.54	19.30 ± 3.06	18.32 ± 4.23	12.73 ± 2.38	10.38 ± 2.42	87.80 ± 12.44
	[25.96–28.19]	[18.55–20.05]	[17.28–19.36]	[12.14–13.31]	[9.78–10.97]	[84.74–90.86]
Good (n = 100)	27.83 ± 3.99	19.52 ± 2.88	18.15 ± 3.99	13.05 ± 2.12	10.82 ± 2.23	89.37 ± 10.14
	[27.04–28.62]	[18.95–20.09]	[17.36–18.94]	[12.63–13.47]	[10.38–11.26]	[87.36–91.38]
High (n = 87)	28.99 ± 3.83	19.86 ± 3.13	19.22 ± 3.31	13.36 ± 1.74	11.36 ± 2.20	92.78 ± 9.27
	[28.17–29.81]	[19.20-20.53]	[18.51–19.92]	[12.99–13.73]	[10.89–11.83]	[90.81–94.76]
	P= 0.042*	P= 0.156	P= 0.353	P= 0.535	P= 0.057	P= 0.028*
**Resided in another country** ^**$**^						
Yes (n = 97)						
	28.93 ± 3.68	19.79 ± 2.65	19.56 ± 3.51	13.46 ± 1.50	11.16 ± 2.34	92.91 ± 8.44
No (n = 175)	[28.19–29.67]	[19.26–20.33]	[18.85–20.26]	[13.16–13.77]	[10.69–11.64]	[91.21–94.61]
	27.59 ± 4.33	19.36 ± 3.14	18.02 ± 4.05	12.82 ± 2.34	10.80 ± 2.27	88.59 ± 11.55
	[26.94–28.23]	[18.89–19.83]	[17.42–18.63]	[12.47–13.17]	[10.46–11.14]	[86.87–90.32]
	P= 0.011*	P= 0.479	P= 0.005*	P= 0.110	P= 0.242	P= 0.003*
**Residing duration** ^¶^	r= 0.107	r= 0.105	r= 0.169	r= 0.025	r= -0.107	r= 0.099
	P= 0.336	P= 0.334	P= 0.126	P= 0.820	P= 0.336	P= 0.374
**Tourism** ^**$**^						
Yes (n = 102)	28.40 ± 4.21	19.56 ± 3.14	19.43 ± 3.76	13.26 ± 2.31	11.11 ± 2.45	91.76 ± 11.29
	[27.58–29.23]	[18.94–20.18]	[18.69–20.17]	[12.81–13.72]	[10.63–11.59]	[89.55–93.98]
No (n = 170)	27.86 ± 4.12	19.49 ± 2.89	18.05 ± 3.95	12.92 ± 1.96	10.82 ± 2.19	89.15 ± 10.29
	[27.24–28.49]	[19.05–19.93]	[17.46–18.65]	[12.63–13.22]	[10.49–11.16]	[87.60-90.71]
	P= 0.234	P= 0.602	P= 0.004*	P= 0.026*	P= 0.303	P= 0.020*
**Tourism duration** ^¶^	r= 0.119	r= 0.347	r= 0.131	r= 0.145	r= 0.186	r= 0.296
	P= 0.390	P= 0.010*	P= 0.345	P= 0.297	P= 0.178	P= 0.030*
**Friendships** ^**$**^						
Yes (n = 217)	28.70 ± 3.88	19.66 ± 2.98	19.27 ± 3.57	13.21 ± 2.02	11.13 ± 2.26	91.96 ± 9.79
	[28.18–29.21]	[19.26–20.06]	[18.79–19.74]	[12.94–13.48]	[10.83–11.43]	[90.65–93.27]
No (n = 55)	25.58 ± 4.29	18.95 ± 2.93	15.82 ± 4.10	12.42 ± 2.28	10.15 ± 2.29	82.91 ± 11.31
	[24.42–26.74]	[18.15–19.74]	[14.71–16.93]	[11.80-13.03]	[9.53–10.76]	[79.85–85.97]
	P < 0.001*	P= 0.154	P < 0.001*	P= 0.010*	P= 0.007*	P < 0.001*
**Neighbors** ^**$**^						
Yes (n = 133)	28.63 ± 4.09	19.47 ± 3.14	19.86 ± 3.38	13.23 ± 2.18	11.38 ± 2.31	92.59 ± 10.44
	[27.93–29.33]	[18.93–20.01]	[19.28–20.45]	[12.86–13.61]	[10.99–11.78]	[90.80-94.38]
No (n = 139)	27.53 ± 4.16	19.55 ± 2.81	17.33 ± 4.01	12.88 ± 2.00	10.50 ± 2.19	87.78 ± 10.52
	[26.83–28.22]	[19.08–20.02]	[16.66–18.01]	[12.54–13.21]	[10.13–10.87]	[86.02–89.55]
	P = 0.017*	P= 0.976	P < 0.001*	P= 0.045*	P= 0.002*	P < 0.001*
**Cultural events** ^**$**^						
Yes (n = 168)	28.68 ± 3.94	19.66 ± 2.98	19.49 ± 3.53	13.16 ± 2.05	11.05 ± 2.27	92.04 ± 9.97
	[28.08–29.28]	[19.21–20.12]	[18.95–20.03]	[12.85–13.47]	[10.71–11.40]	[90.52–93.56]
No (n = 104)	27.08 ± 4.31	19.28 ± 2.97	17.09 ± 4.09	12.88 ± 2.16	10.73 ± 2.32	87.05 ± 11.24
	[26.24–27.92]	[18.70-19.87]	[16.29–17.88]	[12.45–13.30]	[10.28–11.18]	[84.86–89.23]
	P = 0.002*	P= 0.339	P < 0.001*	P= 0.269	P= 0.312	P < 0.001*

SD: standard deviation, CI: Confidence interval

**$**: Mann-Whitney test was performed, **‡**: Kruskal Wallis test was performed ¶: Spearman’s test was performed, **¥**: only mean is reported, as *n* = 1 for Saudi

* Denotes statistically significant difference at 0.05 level

Statistically significant differences were observed across several variables. International students consistently scored higher than domestic students across most subdimensions, particularly in interaction confidence and total ISS (*P* < 0.001 and *P* = 0.001, respectively). Females had significantly higher scores in respect for cultural differences (*P* = 0.001). A positive correlation was observed between age and interaction confidence, attentiveness and total ISS.

Other notable correlates included plurilingualism, higher self-rated English proficiency, and participation in intercultural experiences (e.g., tourism, friendships, neighbors, and cultural events), all of which were associated with significantly higher ISS scores across various subscales (*P* < 0.05). For instance, students with intercultural friendships reported notably higher total ICS scores (mean: 91.96 vs. 82.91, *P* < 0.001).

### Multicultural student group work-related challenges (objective 3)

Table 2 compares the perceived MCSG work-related challenges between domestic and international students. While most challenge scores were similar between groups, domestic students reported significantly more difficulty with culturally different standards of interaction (*P* = 0.004), decision-making styles (*P* = 0.017), conflict management (*P* = 0.025), attitudinal issues (*P* = 0.012), and dominance in group settings (*P* = 0.005). The total challenge score was also slightly higher among domestic students (*P* = 0.023). Other challenge areas, including motivation and communication, showed no significant group differences.

### Perceived value in intercultural group work (objective 4)

Table 3 compares domestic and international students’ perceived value in diversity in intercultural group work. Domestic students reported significantly higher scores across all four items, including learning benefits, skill development, and knowledge enrichment (all *P* ≤ 0.001). The total perceived value score was also notably higher among domestic students (*P* < 0.001).

**Table 2:** Comparison of multicultural student group work related challenges scores between domestic and international students (*n* = 272)

Challenge	Domestic (*n* = 170)		International (*n* = 102)		Significance Level
	Mean ± SD	95% CI for Mean	Mean ± SD	95% CI for Mean	
1. Heterogeneous group composition (grouping students of various ages, genders, and cultures)	2.82 ± 1.34	2.62–3.03	2.82 ± 1.26	2.58–3.07	0.994
2. Differences in content knowledge	3.12 ± 1.11	2.95–3.29	3.04 ± 1.35	2.78–3.31	0.647
3. Differences in academic attitude	3.65 ± 1.15	3.48–3.83	3.37 ± 1.34	3.11–3.64	0.088
4. Difference in ambitions	2.96 ± 1.33	2.76–3.17	3.05 ± 1.36	2.79–3.33	0.607
5. Diverse educational backgrounds	2.99 ± 1.22	2.80–3.17	3.07 ± 1.35	2.80–3.33	0.663
6. Students not communicating properly with fellow students and a supervisor	3.95 ± 1.23	3.77–4.14	3.95 ± 1.19	3.72–4.19	0.933
7. Culturally different standards of interaction	3.16 ± 1.15	2.98–3.33	2.75 ± 1.21	2.52–2.99	0.004*
8. Insufficient English language skills	3.25 ± 1.30	3.05–3.44	3.44 ± 1.33	3.18–3.70	0.214
9. The pressure to defend a group decision while not agreeing with it	3.22 ± 1.19	3.05–3.41	3.25 ± 1.22	3.01–3.50	0.930
10. Culturally different styles of decision-making and problem-solving	3.35 ± 1.17	3.17–3.53	3.01 ± 1.19	2.77–3.25	0.017*
11. Culturally different styles of complying with supervisor’s guidelines	3.22 ± 1.25	3.03–3.41	2.92 ± 1.26	2.67–3.17	0.054
12. Ineffective group work management	4.07 ± 1.14	3.90–4.25	3.85 ± 1.22	3.61–4.09	0.114
13. Culturally different styles of conflict management	3.54 ± 1.14	3.37–3.71	3.19 ± 1.23	2.95–3.44	0.025*
14. Attitudinal problems (dislike, mistrust, lack of cohesion)	4.00 ± 1.21	3.82–4.18	3.55 ± 1.39	3.28–3.83	0.012*
15. Free riding	4.17 ± 1.12	4.00-4.34	4.04 ± 1.11	3.82–4.26	0.269
16. Low level of motivation	3.82 ± 1.15	3.65-4.00	4.02 ± 1.14	3.80–4.24	0.125
17. Dominating group members	4.11 ± 1.10	3.94–4.27	3.64 ± 1.34	3.37–3.90	0.005*
**Total challenges**	59.43 ± 11.00	57.76–61.10	57.01 ± 10.18	55.10-59.01	0.023*

SD: standard deviation, CI: Confidence interval

* Denotes statistically significant difference at 0.05 level

**Table 3:** Comparison of perceived value in diversity between domestic and international students (*n* = 272)

Perceived value in diversity	Domestic (*n* = 170)		International (*n* = 102)		Significance level
	Mean ± SD	95% CI for Mean	Mean ± SD	95% CI for Mean	
1. Benefiting from interacting with the group members.	4.20 ± 1.26	4.06–4.34	3.68 ± 0.92	3.43–3.93	***P*** = 0.001*
2. Group members’ perspectives lead to learning something new.	4.26 ± 1.38	4.13–4.40	3.59 ± 0.90	3.32–3.86	***P*** < 0.001*
3. The teamwork experience is helpful to develop collaboration skills to work in an inter-national context.	4.49 ± 1.24	4.38–4.59	3.90 ± 0.69	3.66–4.15	***P*** < 0.001*
4. Interacting with the other group members enriches knowledge and understanding.	4.36 ± 1.33	4.24–4.48	3.72 ± 0.77	3.45–3.98	***P*** < 0.001*
**Total Perceived value in diversity**	17.31 ± 2.69	16.90 -17.72	14.88 ± 4.41	14.01–15.75	***P*** < 0.001*

SD: standard deviation, CI: Confidence interval

* Denotes statistically significant difference at 0.05 level

### Correlation between intercultural sensitivity and perceived value in diversity (objective 5)

Table 4 shows that perceived value in diversity was positively and significantly correlated with several subdimensions of ISS. The strongest correlations were with interaction engagement (*r* = 0.375, *P* < 0.001) and respect for cultural differences (*r* = 0.349, *P* < 0.001), followed by the total ISS score (*r* = 0.333, *P* < 0.001). A weaker but still significant association was found with interaction enjoyment (*r* = 0.196, *P* = 0.001). No significant correlations were observed for interaction confidence or attentiveness.

**Table 4:** Spearman’s correlation between intercultural sensitivity scale scores and perceived value in diversity

Intercultural Sensitivity Scale	Perceived value in diversity
Interaction engagement subdimension	*r* = 0.375, ***P*** < 0.001*
Respect for cultural differences subdimension	*r* = 0.349, ***P*** < 0.001*
Interaction confidence subdimension	*r* = 0.066, *P* = 0.281
Interaction enjoyment subdimension	*r* = 0.196, ***P*** = 0.001*
Interaction attentiveness subdimension	*r* = 0.098, *P* = 0.107
**Total intercultural sensitivity score**	*r* = 0.333, ***P*** < 0.001*

* Denotes statistically significant difference at 0.05 level

### Correlation between multicultural student group work-related challenges and perceived value in diversity (objective 6)

Table 5 presents the correlation between MCSG work-related challenges and students’ perceived value in diversity. Although most correlations were non-significant, higher perceived value in diversity was weakly and positively correlated with challenges such as ineffective group work management (*r* = 0.172, *P* = 0.005), attitudinal problems (*r* = 0.246, *P* < 0.001), free-riding (*r* = 0.141, *P* = 0.020), and dominating group members (*r* = 0.178, *P* = 0.003). Conversely, a weak negative correlation was found with heterogeneous group composition (*r* = -0.130, *P* = 0.032).

**Table 5:** Correlation between multicultural student group Work-Related challenges scores and perceived value in diversity

Challenge	Perceived Value in Diversity
1. Heterogeneous group composition (diversity in age, gender, and culture)	*r* = -0.130, ***P*** = 0.032*
2. Differences in content knowledge	*r* = -0.039, *P* = 0.529
3. Differences in academic attitude	*r* = 0.018, *P* = 0.768
4. Difference in ambitions	*r* = -0.053, *P* = 0.388
5. Diverse educational backgrounds	*r* = -0.028, *P* = 0.650
6. Students not communicating properly with fellow students and a supervisor	*r* = 0.059, *P* = 0.333
7. Culturally different standards of interaction	*r* = -0.001, *P* = 0.986
8. Insufficient English language skills	*r* = 0.097, *P* = 0.111
9. The pressure to defend a group decision while not agreeing with it	*r* = 0.040, *P* = 0.511
10. Culturally different styles of decision-making and problem-solving	*r* = 0.089, *P* = 0.142
11. Culturally different styles of complying with supervisor’s guidelines	*r* = 0.061, *P* = 0.315
12. Ineffective group work management	*r* = 0.172, ***P*** = 0.005*
13. Culturally different styles of conflict management	*r* = 0.078, *P* = 0.198
14. Attitudinal problems such as dislike, mistrust, and lack of cohesion	*r* = 0.246, ***P*** < 0.001*
15. Free-riding	*r* = 0.141, ***P*** = 0.020*
16. A low level of motivation	*r* = 0.093, *P* = 0.128
17. Dominating group members	*r* = 0.178, ***P*** = 0.003*
**Total challenges score**	*r* = 0.098, *P* = 0.105

* Denotes statistically significant difference at 0.05 level

## Discussion

The current study assessed ICS among third-year national program medical students at AFM, examining various subdimensions of ISS and comparing scores with other studies. The findings suggest that the students generally reported high ICS levels, consistent with studies conducted in Turkey [11] and the United Arab Emirates [8] but differed from findings in Thailand [13], where students scored higher, and Belarus and Poland [14], where scores were notably lower. These differences may reflect cultural contexts, with the collectivist nature of Arab and Eastern societies potentially contributing to higher ICS scores compared to European countries [15].

Several correlates of ICS were identified. The results suggest the nuanced influence of age, prior experiences, and geographical context on the development of ICS among medical students. These results were supported by a recent systematic review [16] that categorized individual-level determinants of ICS into four domains: experiences, cultural values, demographics, and language proficiency. The present study supports this framework, emphasizing how diverse life experiences, demographic characteristics (e.g., age and gender), and language-related factors, particularly English proficiency and plurilingualism, jointly shape students’ ICS.

International students, who likely gained multicultural exposure through their studies in Egypt, reported significantly higher total ISS scores and outperformed domestic students across most subdimensions. This supports the notion that exposure to different cultures contributes to the development of intercultural skills. Since all international students in this study were, by definition, studying abroad, the present study’s findings align with those of a meta-analysis showing that study abroad programs are associated with small to moderate improvements in intercultural competence (effect size g = 0.38) [17], of which ICS is a recognized component [9].

Additionally, female participants in the present study demonstrated higher scores in respect for cultural differences. This finding is consistent with previous studies conducted in Thailand [13], Taiwan [18], and the Midwest of the United States [19], which reported higher ICS levels among females. These results may be attributed to a tendency among females toward empathy, collaboration, and lower tolerance for conflict and aggressiveness. A study from Spain similarly supported this trend [20]. However, the present study did not find significant gender differences across other ISS subdimensions, contrasting with findings from Nepal [21] where males showed higher overall ICS. These inconsistencies underscore the complex interaction between gender and cultural context in shaping ICS among medical students.

Regarding age, the study found positive correlations between older age and higher scores in interaction confidence, interaction attentiveness, and total ISS. This suggests that older students may have developed greater ICS through accumulated life experiences, which enhanced their self-confidence and ability to interpret nuanced social cues. This finding aligns with research from Spain [20] where final-year university students demonstrated better ICS compared to their younger counterparts.

Interestingly, the present study found that domestic students from rural areas exhibited higher interaction attentiveness than their urban counterparts. This may be due to rural students’ familiarity with navigating varying social norms and expectations, which could foster greater attentiveness in intercultural interactions, particularly within the more diverse urban context of Alexandria.

The study also highlighted the significant association between prior education in international schools, self-rated English proficiency, plurilingualism and ICS levels. Participants who had attended international schools before university, rated their English proficiency as excellent, and were plurilingual demonstrated higher ISS scores, especially in subdimensions such as interaction engagement, interaction confidence, and interaction attentiveness. These findings may be attributed to the teaching methods and multicultural curricula typically employed in international schools, which encourage openness and confidence in cross-cultural communication. Moreover, improved language proficiency has been linked to better social interaction skills and confidence in intercultural settings, as noted in studies from Spain [22] and China [23, 24]. The higher attentiveness observed among plurilingual students in the current study suggests that the ability to speak multiple languages enhances sensitivity to nuanced communication cues, such as idioms, tone, and multiple meanings, thereby enriching ICS. These findings support the value of encouraging language development, particularly English proficiency and plurilingualism, as essential components of educational strategies aimed at fostering intercultural competence and preparing students to engage effectively in diverse cultural environments.

The study’s findings regarding the correlation of various factors with ICS offered valuable insights into how different experiences and environments shape students’ abilities to engage effectively with diverse cultures. Notably, having parents from different cultural backgrounds was not significantly associated with students’ ICS levels. However, students whose parents demonstrated higher ICS themselves reported greater total ISS scores and higher interaction engagement. This aligns with findings from a qualitative study in China [25], which suggested that parental role modeling of intercultural competencies can positively influence their children’s sensitivity to other cultures.

Additionally, participants with overseas experiences exhibited higher total ISS scores and outperformed their peers in several ISS subdimensions. These results are consistent with both qualitative [25, 26] and quantitative studies [18, 20, 27, 28], indicating that exposure to different cultures, whether through travel, study abroad programs, or other international experiences, can enhance ICS. However, contradictory findings by Chen et al. [23] underscore the complexity of these relationships and highlight the need for further research to explore the nuanced impact of overseas experiences on the development of ICS.

Having friends and neighbors from different cultures, as well as attending cultural events, was associated with higher total ISS scores and scores in several subdimensions. This finding is supported by studies from Korea [27] and by Pastena et al. [22], both of which emphasized the role of multicultural friendships and interactions in fostering ICS. These results suggest that educational institutions should actively promote intercultural engagement through shared classes, elective courses, and extracurricular cultural events. Such efforts enhance domestic students’ ICS through “internationalization-at-home,” a concept supported by an experimental study [29]. These initiatives may also benefit international students by increasing opportunities for meaningful contact with host nationals, as demonstrated in a recent study [30].

The findings on MCSG work-related challenges among domestic and international students provided further insight into how cultural and educational backgrounds shape perceptions of collaboration. Both groups identified free-riding as the most significant MCSG work-related challenge, consistent with previous research [10]. Interestingly, despite the collectivist orientation of Egyptian and Arab societies, domestic students rated free-riding as a greater concern than international students. This may be attributed to the highly competitive nature of medical education in Egypt, where academic success is strongly emphasized. In contrast, international students may have come from educational systems with different approaches to collaboration and assessment.

Moreover, domestic students rated culturally different standards of interaction, decision-making, and dealing with dominating group members as more challenging than their international peers. These perceptions may reflect domestic students’ stronger familiarity and comfort with their own cultural norms and expectations. Conversely, international students, having navigated more diverse cultural environments, might be more adaptable in multicultural group settings.

To address the MCSG work-related challenges perceived in work, the current study suggests several strategies. Ensuring fair evaluation rubrics that individualize marks can reduce concerns about free-riding. Empowering students with communication skills to navigate conflicts and diverse group dynamics can also help alleviate challenges related to interaction norms, decision making, and group dynamics. Nonetheless, as the study did not incorporate qualitative follow-up methods such as interviews or open-ended responses, the underlying reasons and contextual factors behind the reported MCSG work-related challenges remain insufficiently understood.

The current study explored the perceived value of diversity among domestic and international students, and its correlation with ICS and MCSG work-related challenges. Domestic students reported a higher appreciation for diversity compared to international peers, contrasting with findings from the Netherlands [7], where the opposite pattern was observed. Understanding the basis for these differing perceptions may help inform strategies to enhance inclusive learning environments and foster effective intercultural collaboration.

The findings revealed that the perceived value in diversity was positively correlated with several subdimensions of the ISS. This suggests that students who enjoy engaging with, and respect other cultures are more likely to perceive multicultural interactions as valuable. In turn, recognizing the potential benefits of such interactions may further enhance students’ engagement and openness toward culturally diverse peers. Conversely, several MCSG work-related challenges were only weakly correlated with perceived value, implying that students may still appreciate the growth opportunities offered by multicultural collaboration despite facing difficulties.

The internal consistency of the Arabic-translated ISS subdimensions varied. While the overall ISS scale demonstrated good reliability (α = 0.836), some subscales, such as interaction attentiveness (α = 0.443) and respect for cultural differences (α = 0.574), showed low Cronbach’s alpha values. These lower reliabilities may reflect linguistic or cultural nuances in how specific items are interpreted in the Arabic context, rather than inherent flaws in the constructs themselves. This highlights the importance of further cultural adaptation and validation of the ISS for Arabic-speaking populations. Future research should focus on re-validating the scale, revising or replacing weaker items, and employing confirmatory factor analysis and cognitive interviewing during translation to enhance the psychometric robustness and cultural relevance of the instrument.

Although studies on ICS among Arab medical students are lacking, comparisons with international contexts can help situate the present findings. In Nepal, over two-thirds of medical students reported a good to excellent attitude toward cultural diversity, with males and older students scoring significantly higher. This aligned with our findings regarding age but contrasted with our result that females scored higher in certain ISS subdimensions [21]. In Iran, a study showed that medical students from four ethnic groups showed moderate to high scores in all dimensions of ISS [31], supporting our observation of generally high ISS scores. In Chile, interestingly, first-year medical students scored higher in ICS than final-year students [32], possibly due to recent intercultural exposure in high school, which differed from our context where older students demonstrated higher scores. These comparisons, while not from the Arab world, underscore the novelty of the present study and highlight the need for more region-specific research among Arab medical students.

The current study presents several strengths. This study is the first of its kind on ICS in Egypt and the second among university students in the Arab world [8]. It also adds to the limited research on medical students in the Arab world and Eastern Mediterranean Region, providing valuable insights into a previously understudied population. This comprehensive approach allows for a detailed analysis of ICS among different nationalities within the same educational context. The investigated correlates such as the pre-university school system (international vs. national), has never been previously explored in similar contexts and studies. Additionally, it correlated ICS with MCSG work-related challenges and perceived value in intercultural group work, contributing to a more holistic understanding of intercultural dynamics among students. However, some limitations are documented. The study employed a quota-convenience sampling method, which, while ensuring proportional representation of domestic and international students, may limit the generalizability of the findings. As participants were not randomly selected, the sample may not accurately reflect the full diversity of third-year medical students at AFM or in similar institutions. Students who chose to respond may have had stronger opinions or greater interest in intercultural issues, introducing self-selection bias. This method also increases the risk of selection bias, as certain subgroups, such as less engaged students or those with limited internet access, may have been underrepresented. Consequently, the findings should be interpreted as context-specific and exploratory rather than broadly generalizable. Additionally, the study did not incorporate qualitative follow-up methods such as interviews or open-ended questions. This limits the depth of understanding regarding the specific contexts and underlying reasons behind the reported MCSG work-related challenges. Including qualitative insights could have provided richer interpretations of students’ experiences. English proficiency and parents’ ICS were self-reported, which may introduce inaccuracies due to participants’ subjective perceptions or biases. Participants may have responded in a socially desirable manner, particularly in assessments of ISS and perceived value in diversity, which could influence the validity of the findings. Proficiency in Arabic dialects, which could act as a communication barrier between different nationalities, was not investigated. The low Cronbach’s Alpha for some subdimensions of the Arabic version of ISS indicates potential reliability issues with the measurement instrument, which could affect the consistency and accuracy of the study’s results. Due to the cross-sectional design, causal relationships cannot be inferred between variables measured at a single point in time, limiting the ability to draw conclusions about the direction of relationships observed.

## Conclusion

This study provides valuable insights into ICS among third-year national program medical students at AFM, suggesting a potential link with language proficiency, multicultural exposure, and prior educational background. Overall, students reported relatively high ICS levels. While domestic students reported more MCSG work-related challenges, they also perceived greater value in such experiences, suggesting a complex relationship between perceived difficulties and perceived benefits. However, due to the use of non-probability sampling, self-reported measures, and the context-specific nature of the study, the findings may not be generalizable to all medical students or institutions. Future research using representative samples and conducted in diverse educational settings is needed to further explore these dynamics.

## Electronic supplementary material

Below is the link to the electronic supplementary material.

Supplementary Material 1

Supplementary Material 2

Supplementary Material 3

Supplementary Material 4

## Data Availability

The datasets used and/or analysed during the current study are available from the corresponding author on reasonable request.

## Abbreviations

AFM: Alexandria Faculty of Medicine
ICS: Intercultural sensitivity
ISS: Intercultural sensitivity scale
MCSG: Multicultural student group
