# Supplementary material for: Intercultural sensitivity, challenges, and perceived value in multicultural group work among third-year medical students at Alexandria university, Egypt (2023–2024)
Source: BMC Med Educ. 2025 Jul 11;25:1038. doi: 10.1186/s12909-025-07597-7 (PMC12255028; doi:10.1186/s12909-025-07597-7)
Supplement: Supplementary file 1 — Supplementary Material 1 [file 12909_2025_7597_MOESM1_ESM.docx]

**Intercultural Sensitivity, Challenges, and Perceived Value in Multicultural Group Work Among Third-Year Medical Students at Alexandria University, Egypt (2023)**

The world is increasingly interconnected, and we often interact with people from diverse cultures. Positive and appreciative intercultural interactions are essential for achieving the best outcomes. This is especially evident in medical care, where effective communication and teamwork across different cultures play a critical role.

This study aims to assess the level of intercultural sensitivity, which is the ability to understand and appreciate differences between cultures to improve communication between them. It is conducted among third year National program medical students, at Alexandria Faculty of Medicine, to determine the factors that affect their level of intercultural sensitivity. Additionally, it examines inherent challenges in multicultural student group work and the percieved value in diversity, with the goal of enhancing the cultural competence of medical graduates.

The questionnaire is divided into four parts:

- Part 1: General characteristcs of the participants.

- Part 2: Intercultural sensitivity scale.

- Part 3: Challenges of multicultural student group work.

- Part 4: Percieved value in diversity.

In the context of this study, culture is the system of shared beliefs, values, and practices among group members to understand, deal with, and interact with each other and their environment, which is transmitted from generation to generation through sharing and learning

Your participation is completely optional and voluntary. You have the right not to participate in the study, and you may withdraw at any time, without any negative consequences for you if you do not participate or withdraw.

None of these questions trace back to your personal identity. Your information and data will remain completely confidential, and only researchers and those conducting the study will be able to access it.

- I agree to participate in the study, on a voluntary basis.

- I do not agree to participate in the study.

**Intercultural Sensitivity, Challenges, and Perceived Value in Multicultural Group Work Among Third-Year Medical Students at Alexandria University, Egypt (2023)**

**(Non-Egyptian/ International students’ version)**

**Part 1: General characteristics:**

1. Age: ___ years.

2. Gender: ( ) Male.

( ) Female.

3. Nationality: ( ) Jordanian. ( ) Sudanese.

( ) Palestinian. ( ) Saudi Arabian.

( ) Syrian. ( ) Bahraini

( ) Iraqi. ( ) Mauritanian

( ) Yemeni. ( ) Libyan.

4. Are you still in touch with your home culture? ( ) Yes.

( ) No.

5. Your pre-university (school) education was: ( ) National

( ) International

( ) National for some years

and international for others.

6. How do you evaluate your English proficiency? ( ) Poor.

( ) Moderate.

( ) Good.

( ) Excellent

7. Are you fluent in a language other than Arabic (and English)? ( ) Yes.

( ) No.

8. Do your parents descend from different cultures? ( ) Yes.

( ) No.

9. How do you evaluate your parents’ intercultural sensitivity?

( ) Weak (they don’t identify nor appreciate the differences between cultures).

( ) Moderate.

( ) Good.

( ) High (they identify and appreciate the differences between cultures).

10. Growing up, have you lived in a country (other than the one whose nationality you carry, and other than Egypt) prior to entering the university?

( ) Yes, I lived in: _________________ for _____ years.

( ) No.

11. Have you visited (as a tourist) countries other the one whose nationality you carry, where you grew up, and Egypt?

( ) Yes, I visited: ___________, __ times and spent a total of __ months.

( ) No.

12. Do you have friends from other cultures? ( ) Yes.

( ) No.

13. Do you have neighbors from other cultures? ( ) Yes.

( ) No.

14. Have you attended an event from a culture other than your own? ( ) Yes.

( ) No.

**Part 2: Intercultural Sensitivity:**

Below is a series of statements concerning
intercultural communication. There are no right or
wrong answers. Please work quickly and record your
first impression by indicating the degree to which you
agree or disagree with the statement, where
1= Strongly disagree, and 5= Strongly agree.

1= Strongly agree

5= Strongly agree

4= Agree

2= Disagree

3= Uncertain

| 5 | 4 | 3 | 2 | 1 | 1. I enjoy interacting with people from different cultures. |
| --- | --- | --- | --- | --- | --- |
| 5 | 4 | 3 | 2 | 1 | 2. I think people from other cultures are narrow-minded. |
| 5 | 4 | 3 | 2 | 1 | 3. I am pretty sure of myself in interacting with people from different cultures. |
| 5 | 4 | 3 | 2 | 1 | 4. I find it very hard to talk in front of people from different cultures. |
| 5 | 4 | 3 | 2 | 1 | 5. I always know what to say when interacting with people from different cultures. |
| 5 | 4 | 3 | 2 | 1 | 6. I can be as sociable as I want to be when interacting with people from different cultures. |
| 5 | 4 | 3 | 2 | 1 | 7. I don't like to be with people from different cultures. |
| 5 | 4 | 3 | 2 | 1 | 8. I respect the values of people from different cultures. |
| 5 | 4 | 3 | 2 | 1 | 9. I get upset easily when interacting with people from different cultures. |
| 5 | 4 | 3 | 2 | 1 | 10. I feel confident when interacting with people from different cultures. |
| 5 | 4 | 3 | 2 | 1 | 11. I tend to wait before forming an impression of culturally-distinct counterparts. |
| 5 | 4 | 3 | 2 | 1 | 12. I often get discouraged when I am with people from different cultures. |
| 5 | 4 | 3 | 2 | 1 | 13. I am open-minded to people from different cultures. |
| 5 | 4 | 3 | 2 | 1 | 14. I am very observant when interacting with people from different cultures. |
| 5 | 4 | 3 | 2 | 1 | 15. I often feel useless when interacting with people from different cultures. |
| 5 | 4 | 3 | 2 | 1 | 16. I respect the ways people from different cultures behave. |
| 5 | 4 | 3 | 2 | 1 | 17. I try to obtain as much information as I can when interacting with people from different cultures. |
| 5 | 4 | 3 | 2 | 1 | 18. I would not accept the opinions of people from different cultures. |
| 5 | 4 | 3 | 2 | 1 | 19. I am sensitive to my culturally-distinct counterpart's subtle meanings during our interaction. |
| 5 | 4 | 3 | 2 | 1 | 20. I think my culture is better than other cultures. |
| 5 | 4 | 3 | 2 | 1 | 21. I often give positive responses to my culturally-different counterpart during our interaction. |
| 5 | 4 | 3 | 2 | 1 | 22. I avoid those situations where I will have to deal with culturally-distinct persons. |
| 5 | 4 | 3 | 2 | 1 | 23. I often show my culturally-distinct counterpart my understanding through verbal or nonverbal cues. |
| 5 | 4 | 3 | 2 | 1 | 24. I have a feeling of enjoyment towards differences between my culturally-distinct counterpart and me. |

**Part 3 and 4: Multicultural student group work**

Throughout your past university years, there was direct interaction with your International colleagues in the National program. You experienced multicultural group work in assignments. The third and fourth parts of the questionnaire aim to answer two questions: What challenges did you face as you worked in a multicultural group? And what value do you see in multicultural group work and diversity?

**Part 3: Challenges that faced multicultural student group work:**

Below is a series of statements concerning
challenges that may face multicultural student
group work. Rate each one according to the extent
you perceived it caused a challenge when you
worked multicultural group, where 1 = it was
very unimportant (not to worry about), and
5 = it was a very important challenge to consider.

1= Very unimportant

5= Very important

4= Important

2= Unimportant

3= Neutral

| 5 | 4 | 3 | 2 | 1 | 1. Heterogeneous group composition (grouping students of a variety of age, gender and culture). |
| --- | --- | --- | --- | --- | --- |
| 5 | 4 | 3 | 2 | 1 | 2. Differences in content knowledge. |
| 5 | 4 | 3 | 2 | 1 | 3. Differences in academic attitude. |
| 5 | 4 | 3 | 2 | 1 | 4. Difference in ambitions. |
| 5 | 4 | 3 | 2 | 1 | 5. Diverse educational backgrounds. |
| 5 | 4 | 3 | 2 | 1 | 6. Students not communicating properly with fellow students and a supervisor. |
| 5 | 4 | 3 | 2 | 1 | 7. Culturally different standards of interaction. |
| 5 | 4 | 3 | 2 | 1 | 8. Insufficient English language skills. |
| 5 | 4 | 3 | 2 | 1 | 9. The pressure to defend a group decision while not agreeing with it. |
| 5 | 4 | 3 | 2 | 1 | 10. Culturally different styles of decision making and problem solving. |
| 5 | 4 | 3 | 2 | 1 | 11. Culturally different styles of complying with supervisor's guidelines |
| 5 | 4 | 3 | 2 | 1 | 12. Ineffective group work management |
| 5 | 4 | 3 | 2 | 1 | 13. Culturally different styles of conflict management |
| 5 | 4 | 3 | 2 | 1 | 14. Attitudinal problems such as dislike, mistrust and lack of cohesion. |
| 5 | 4 | 3 | 2 | 1 | 15. Free-riding. |
| 5 | 4 | 3 | 2 | 1 | 16. A low level of motivation. |
| 5 | 4 | 3 | 2 | 1 | 17. Dominating group members |

**Part 4: Perceived value in diversity:**

Below is a series of statements concerning the
percieved value in diversity. There are no right
or wrong answers. Please work quickly and record
your first impression by indicating the degree to which
you agree or disagree with the statement,
where 1= Strongly disagree, and 5= Strongly agree.

1= Strongly agree

5= Strongly agree

4= Agree

2= Disagree

3= Uncertain

| 5 | 4 | 3 | 2 | 1 | 1. I have benefited from interacting with the group members. |
| --- | --- | --- | --- | --- | --- |
| 5 | 4 | 3 | 2 | 1 | 2. Group members’ perspective led me to learn something new. |
| 5 | 4 | 3 | 2 | 1 | 3. The teamwork experience is helpful to develop my collaboration skills to work in an international context. |
| 5 | 4 | 3 | 2 | 1 | 4. Interacting with other group members enriched my knowledge and understanding. |

**Thank you for your time.**
