# Supplementary material for: Intercultural sensitivity, challenges, and perceived value in multicultural group work among third-year medical students at Alexandria university, Egypt (2023–2024)
Source: BMC Med Educ. 2025 Jul 11;25:1038. doi: 10.1186/s12909-025-07597-7 (PMC12255028; doi:10.1186/s12909-025-07597-7)
Supplement: Supplementary file 3 — Supplementary Material 3 [file 12909_2025_7597_MOESM3_ESM.docx]

**Table 1 (Supplementary material)**

**Cronbach’s Alpha coefficients for the Intercultural Sensitivity Scale and Perceived Value in Intercultural Group Work**

| **Scale/ subdimension** | **No. of Items** | **Cronbach’s α** |
| --- | --- | --- |
| **Intercultural Sensitivity Scale** |  |  |
| - Interaction Engagement | 7 | 0.705 |
| - Respect for Cultural Differences | 6 | 0.574 |
| - Interaction Confidence | 5 | 0.794 |
| - Interaction Enjoyment | 3 | 0.645 |
| - Interaction Attentiveness | 3 | 0.443 |
| - Total Scale | 24 | 0.836 |
| **Perceived value in intercultural group work** | 4 | **0.865** |
