# Supplementary material for: Intercultural sensitivity, challenges, and perceived value in multicultural group work among third-year medical students at Alexandria university, Egypt (2023–2024)
Source: BMC Med Educ. 2025 Jul 11;25:1038. doi: 10.1186/s12909-025-07597-7 (PMC12255028; doi:10.1186/s12909-025-07597-7)
Supplement: Supplementary file 4 — Supplementary Material 4 [file 12909_2025_7597_MOESM4_ESM.docx]

**Table 2 (Supplementary material)**

**General characteristics of the studied population (n=272)**

| **1. Personal characteristics** | **No. (n=272)** | **%** | |
| --- | --- | --- | --- |
| **Attended class**  Egyptian  Foreigner  **Gender**  Male  Female | 170  102  146  126 | 62.5  36.5  53.7  46.3 | |
|  | **Mean ± SD** | | |
| **Age** | 20.5 ± 1.04 | | |
| **Egyptian students** | **No. (n=170)** | **%** | |
| **Residence**  Urban  Rural | 129  41 | 75.9  24.1 | |
| **Foreign students** | **No. (n=102)** | **%** | |
| **Nationality**  Jordanian  Iraqi  Palestinian  Syrian  Sudanese  Yemeni  Mauritanian  Saudi  **Being in touch with home culture**  Yes  No | 28  20  16  15  14  4  4  1  100  2 | 27.5  19.6  15.7  14.7  13.7  3.9  3.9  1.0  98  2 | |
| **2. Educational background** | **No. (n=272)** | **%** | |
| **Education system in school**  National  International  Both national and international  **Perceived English proficiency level**  Poor  Moderate  Good  Excellent  **Plurilingualism**  Yes  No | 210  30  32  8  72  127  65  73  199 | 77.2  11.0  11.8  2.9  26.5  46.7  23.9  26.8  73.2 | |
| **3. Parents** | **No. (n=272)** | **%** | |
| **Parents’ cultural backgrounds**  The same  Different  **Parents’ perceived ICS level**  Poor  Average  Good  High | 259  13  19  66  100  87 | 95.2  4.8  7  24.2  36.8  32.0 | |
| **4. Presence of overseas experiences** | **No. (n=272)** | | **%** |
| **Resided in another country**  Yes  No | 97  175 | | 35.7  64.3 |
| **Visited other countries (tourism)**  Yes  No | 102  170 | | 37.5  62.5 |
| **Duration of overseas experiences** | **Mean ± SD** | | |
| **Years residing in another country** | 14.30 ± 5.04 | | |
| **Months visiting other countries** | 5.01 ± 5.89 | | |
| **5. Multicultural experiences** | **No. (n=272)** | | **%** |
| **Friends from different cultures**  Yes  No  **Neighbors from different cultures**  Yes  No  **Attended cultural events**  Yes  No | 217  55  133  139  168  104 | | 79.8  20.2  48.9  51.1  61.8  38.2 |

SD: standard deviation.
